# Modeling biological rhythms in failure time data

**DOI:** 10.1186/1740-3391-4-14

**Published:** 2006-11-07

**Authors:** Naser B Elkum, James D Myles

**Affiliations:** 1 Department of Biostatistics, Epidemiology, and Scientific Computing, King Faisal Specialist Hospital & Research Center, Riyadh 11211, Saudi Arabia; 2 Clinical Statistics, Pfizer Global Research and Development (PGRD), Ann Arbor Laboratories, Ann Arbor, MI 48105, USA

## Abstract

**Background:**

The human body exhibits a variety of biological rhythms. There are patterns that correspond, among others, to the daily wake/sleep cycle, a yearly seasonal cycle and, in women, the menstrual cycle. Sine/cosine functions are often used to model biological patterns for continuous data, but this model is not appropriate for analysis of biological rhythms in failure time data.

**Methods:**

We adapt the cosinor method to the proportional hazards model and present a method to provide an estimate and confidence interval of the time when the minimum hazard is achieved. We then apply this model to data taken from a clinical trial of adjuvant of pre-menopausal breast cancer patients.

**Results:**

The application of this technique to the breast cancer data revealed that the optimal day for pre-resection incisional or excisional biopsy of 28-day cycle (i. e. the day associated with the lowest recurrence rate) is day 8 with 95% confidence interval of 4–12 days. We found that older age, fewer positive nodes, smaller tumor size, and experimental treatment were predictive of longer relapse-free survival.

**Conclusion:**

In this paper we have described a method for modeling failure time data with an underlying biological rhythm. The advantage of adapting a cosinor model to proportional hazards model is its ability to model right censored data. We have presented a method to provide an estimate and confidence interval of the day in the menstrual cycle where the minimum hazard is achieved. This method is not limited to breast cancer data, and may be applied to any biological rhythms linked to right censored data.

## Background

The human body exhibits a variety of biological rhythms. There are patterns that correspond, among others, to the daily wake/sleep cycle, a yearly seasonal cycle and, in women, the menstrual cycle. The clinical relevance of circadian rhythm has been demonstrated in multi-center randomized trials [1-5]. They have confirmed the clinical findings that optimal timing of chemotherapy can lead to decreased toxicity. Halberg et al. [6] suggested a putative benefit from timing nutriceuticals for preventive or curative health care.

Various mathematical models have been used to assess the suitability of periodic functions associated with biological rhythms. The most common approach is that of "cosinor rhythmometry", in which a linear least squares regression is used to fit a sinusoidal curve to time-series data [7-10]. Tong [7] described the polar coordinate transformation by which the sinusoidal regression problem can be treated as a linear regression problem. Ware and Bowden [8] suggested applying Rao's growth curve analysis to distinguish between inter-and intra-subject variances to draw conclusions about population parameters. Nelson et al [9] have provided a thorough review of calculations and analytic techniques including single, group, and population statistics. Another suggested approach for analysis has been to integrate the sinusoid to account for differences between point- and time-averaged data [10]. Others have considered a nonparametric smooth curve based on fitting a periodic spline function for human circadian rhythms [11,12]. These approaches, however, are intended to study measurements over time from multiple subjects to study the inherent dynamics of circadian rhythms. Although these curve-fitting techniques can be helpful, they are not suitable for right-censored failure time data (FTD).

Failure may be broadly defined as the occurrence of a pre-specified event. Events of this nature include time of death, disease occurrence or recurrence and remission. One important aspect of FTD is that the anticipated event may not occur for each individual under study. This situation is referred to as censoring and the study subject for which no failure time is available is referred to as censored. Censored data analysis requires special methods to compensate for the information lost by not knowing the time of failure of all individuals. The literature is short of methodologies that deal with circadian or biological rhythms in failure time data.

This article models the biological rhythms in censored data. It presents a method to estimate the time that achieves the minimum hazard along with its associated confidence interval. The model is then used to predict the optimal day in the menstrual cycle for breast cancer surgery (i.e. day associated with the lowest recurrence rate) in pre-menopausal women using data from the National Cancer Institute of Canada's Clinical Trial Group MA.5 study.

## Methods

### The Model

The most common approach to the analysis of biological rhythm is that of "cosinor rhythmometry" (see Nelson et al. [9]). Cosinor analysis involves representation of data span by the best-fitting cosine function of the form:

*f*(*t_i_*) = *M *+ *A*cos(*ωt_i _*+ *φ*) + ε_*i *_    (1)

where t_i _represents the time of measurements for the i^th ^individual, M the mean level (termed mesor) of the cosine curve, A is the amplitude of the function, ω is the angular frequency (period) of the curve, and φ is the acrophase (horizontal shift) of the curve. It is assumed that the errors, ε_i_, are independent and normally distributed with means zero and a common residual variance σ^2^. It is also possible to use more than one cosine function with different values of ω (whether or not in harmonic relation) or a combined linear-nonlinear rhythmometry [13,14]. The equation can be fitted to the data by conventional methods of least-squares regression analysis. Obviously, this assumption will not hold for failure time data with skewed and censored observations.

The Cox regression model (proportional hazard model) [15] is the appropriate method for regression analysis of survival data. This model is frequently used to estimate the effect of one or more covariates on a failure time distribution. Let X^T ^= (X_1_, ..., X_*p*_) denote *p *measured covariates on a given individual with censored failure time observation. Then the proportional hazards model can be written as

λ(t|X)=λ0(t)eβTX     (2)
 MathType@MTEF@5@5@+=feaafiart1ev1aaatCvAUfKttLearuWrP9MDH5MBPbIqV92AaeXatLxBI9gBaebbnrfifHhDYfgasaacH8akY=wiFfYdH8Gipec8Eeeu0xXdbba9frFj0=OqFfea0dXdd9vqai=hGuQ8kuc9pgc9s8qqaq=dirpe0xb9q8qiLsFr0=vr0=vr0dc8meaabaqaciaacaGaaeqabaqabeGadaaakeaaiiGacqWF7oaBdaqadaqaaiabdsha0jabcYha8Hqaaiab+HfaybGaayjkaiaawMcaaiabg2da9iab=T7aSnaaBaaaleaacqaIWaamaeqaaOWaaeWaaeaacqWG0baDaiaawIcacaGLPaaacqWGLbqzdaahaaWcbeqaaiab=j7aInaaCaaameqabaGaemivaqfaaSGaemiwaGfaaOGaaCzcaiaaxMaadaqadaqaaiabikdaYaGaayjkaiaawMcaaaaa@4479@

where λ_0_(t) is a baseline hazard corresponding to X^T ^= (0, ..., 0), β^T ^= (β_1_, ..., β_*p*_) is a vector of regression coefficients, and β^T^X is an inner product. The important inference questions in this setting are about the conditional distribution of failure, given the covariates. In order to examine the effect of biological rhythm upon survival, one needs to adapt cosinor rhythmometry to the proportional hazard model.

Let us assume that we have *n *independent individuals (*i *= 1, . . . *n*). For each individual *i*, the survival data consist of the time of the event or the time of censoring ξ_*i*_, an indicator variable, δ_i_, with a value of 1 if ξ_*i *_is uncensored or a value of 0 if ξ_*i *_is censored, and X_*i *_= [*X*_1*i *_*X*_2*i*_]^*T*^, so that the observed data are (ξ_*i*_, δ_i_, *X*_i_). Here *X*_1*i *_= cos*ωt_i_*, *X*_2*i *_= sin*ωt*_*i *_and ω = 2π/τ. Hence *β*_1 _= A cos*φ *and *β*_2 _= - A sin*φ*. The angular frequency (ω) must be set based on our knowledge of the pattern; this is often based on 24 hours but can be different values for different individuals.

The proportional hazards model (2) for the *ith *of *n *individuals can be re-expressed as:

*λ*_*i*_(*t*) = *λ*_0_(*t*)exp(*β*_1_*x*_1*i *_+ *β*_2_*x*_2*i*_)     (3)

This equation is almost the same as the cosinor equations with the same meaning. We should notice that in this model the effects are multiplicative instead of additive. The mesor parameter M is taken up in λ_0_(t), and it is difficult to draw the cosine curve on top of the data as in the continuous case.

### Parameter Estimation

The β-coefficients in the proportional hazards model, which are the unknown parameters in the model, can be estimated using the *method of partial maximum likelihood*. Let *t*_1 _< ... <*t*_*L *_denote the *L *ordered times of observed failures. Let (*i*) provide the case label for the individual failing at *t*_0 _so the covariates associated with the *L *failures are X_(1)_, ..., X_(*L*) _. The log partial likelihood is given by

log⁡ L(β)=∑i=1L[βTX(i)−log⁡{∑j∈ℜiexp⁡(βTXj)}]
 MathType@MTEF@5@5@+=feaafiart1ev1aaatCvAUfKttLearuWrP9MDH5MBPbIqV92AaeXatLxBI9gBaebbnrfifHhDYfgasaacH8akY=wiFfYdH8Gipec8Eeeu0xXdbba9frFj0=OqFfea0dXdd9vqai=hGuQ8kuc9pgc9s8qqaq=dirpe0xb9q8qiLsFr0=vr0=vr0dc8meaabaqaciaacaGaaeqabaqabeGadaaakeaacyGGSbaBcqGGVbWBcqGGNbWzcqqGGaaicqqGmbatdaqadaqaaGGaciab=j7aIbGaayjkaiaawMcaaiabg2da9maaqahabaWaamWaaeaacqWFYoGydaahaaWcbeqaaiabdsfaubaaieaakiab+HfaynaaBaaaleaadaqadaqaaiabdMgaPbGaayjkaiaawMcaaaqabaGccqGHsislcyGGSbaBcqGGVbWBcqGGNbWzdaGadaqaamaaqafabaGagiyzauMaeiiEaGNaeiiCaa3aaeWaaeaacqWFYoGydaahaaWcbeqaaiabdsfaubaakiab+HfaynaaBaaaleaacqWGQbGAaeqaaaGccaGLOaGaayzkaaaaleaacqWGQbGAcqGHiiIZcqGHCeIWdaWgaaadbaGaemyAaKgabeaaaSqab0GaeyyeIuoaaOGaay5Eaiaaw2haaaGaay5waiaaw2faaaWcbaGaemyAaKMaeyypa0JaeGymaedabaGaemitaWeaniabggHiLdaaaa@61D8@

where ℜ_*i *_is the set of cases at risk at time *t*_*i*_. The efficient score for β, Z(β) = ∂/∂β log L(β), is

Z(β)=∑i=1L{X(i)−∑j∈ℜiXjexp⁡(βTXj)∑j∈ℜiexp⁡(βTXj)}     (4)
 MathType@MTEF@5@5@+=feaafiart1ev1aaatCvAUfKttLearuWrP9MDH5MBPbIqV92AaeXatLxBI9gBaebbnrfifHhDYfgasaacH8akY=wiFfYdH8Gipec8Eeeu0xXdbba9frFj0=OqFfea0dXdd9vqai=hGuQ8kuc9pgc9s8qqaq=dirpe0xb9q8qiLsFr0=vr0=vr0dc8meaabaqaciaacaGaaeqabaqabeGadaaakeaacqqGAbGwdaqadaqaaGGaciab=j7aIbGaayjkaiaawMcaaiabg2da9maaqahabaWaaiWaaeaacqqGybawdaWgaaWcbaWaaeWaaeaacqWGPbqAaiaawIcacaGLPaaaaeqaaOGaeyOeI0YaaSaaaeaadaaeqaqaaiabbIfaynaaBaaaleaacqWGQbGAaeqaaOGagiyzauMaeiiEaGNaeiiCaa3aaeWaaeaacqWFYoGydaahaaWcbeqaaiabdsfaubaakiabbIfaynaaBaaaleaacqWGQbGAaeqaaaGccaGLOaGaayzkaaaaleaacqWGQbGAcqGHiiIZcqGHCeIWdaWgaaadbaGaemyAaKgabeaaaSqab0GaeyyeIuoaaOqaamaaqababaGagiyzauMaeiiEaGNaeiiCaa3aaeWaaeaacqWFYoGydaahaaWcbeqaaiabdsfaubaakiabbIfaynaaBaaaleaacqWGQbGAaeqaaaGccaGLOaGaayzkaaaaleaacqWGQbGAcqGHiiIZcqGHCeIWdaWgaaadbaGaemyAaKgabeaaaSqab0GaeyyeIuoaaaaakiaawUhacaGL9baaaSqaaiabdMgaPjabg2da9iabigdaXaqaaiabdYeambqdcqGHris5aOGaaCzcaiaaxMaadaqadaqaaiabisda0aGaayjkaiaawMcaaaaa@6DD6@

Maximum partial likelihood estimates β are found by solving the *p *simultaneous equations Z(β) = 0.

Let β^1
 MathType@MTEF@5@5@+=feaafiart1ev1aaatCvAUfKttLearuWrP9MDH5MBPbIqV92AaeXatLxBI9gBaebbnrfifHhDYfgasaacH8akY=wiFfYdH8Gipec8Eeeu0xXdbba9frFj0=OqFfea0dXdd9vqai=hGuQ8kuc9pgc9s8qqaq=dirpe0xb9q8qiLsFr0=vr0=vr0dc8meaabaqaciaacaGaaeqabaqabeGadaaakeaaiiGacuWFYoGygaqcamaaBaaaleaacqaIXaqmaeqaaaaa@2F80@ and β^2
 MathType@MTEF@5@5@+=feaafiart1ev1aaatCvAUfKttLearuWrP9MDH5MBPbIqV92AaeXatLxBI9gBaebbnrfifHhDYfgasaacH8akY=wiFfYdH8Gipec8Eeeu0xXdbba9frFj0=OqFfea0dXdd9vqai=hGuQ8kuc9pgc9s8qqaq=dirpe0xb9q8qiLsFr0=vr0=vr0dc8meaabaqaciaacaGaaeqabaqabeGadaaakeaaiiGacuWFYoGygaqcamaaBaaaleaacqaIYaGmaeqaaaaa@2F82@ be the partial likelihood estimates of β_1 _and β_2 _respectively. Then, the parameter estimates φ^
 MathType@MTEF@5@5@+=feaafiart1ev1aaatCvAUfKttLearuWrP9MDH5MBPbIqV92AaeXatLxBI9gBaebbnrfifHhDYfgasaacH8akY=wiFfYdH8Gipec8Eeeu0xXdbba9frFj0=OqFfea0dXdd9vqai=hGuQ8kuc9pgc9s8qqaq=dirpe0xb9q8qiLsFr0=vr0=vr0dc8meaabaqaciaacaGaaeqabaqabeGadaaakeaaiiGacuWFgpGzgaqcaaaa@2E7C@ can be obtained by reconverting the estimated β^1
 MathType@MTEF@5@5@+=feaafiart1ev1aaatCvAUfKttLearuWrP9MDH5MBPbIqV92AaeXatLxBI9gBaebbnrfifHhDYfgasaacH8akY=wiFfYdH8Gipec8Eeeu0xXdbba9frFj0=OqFfea0dXdd9vqai=hGuQ8kuc9pgc9s8qqaq=dirpe0xb9q8qiLsFr0=vr0=vr0dc8meaabaqaciaacaGaaeqabaqabeGadaaakeaaiiGacuWFYoGygaqcamaaBaaaleaacqaIXaqmaeqaaaaa@2F80@ and β^2
 MathType@MTEF@5@5@+=feaafiart1ev1aaatCvAUfKttLearuWrP9MDH5MBPbIqV92AaeXatLxBI9gBaebbnrfifHhDYfgasaacH8akY=wiFfYdH8Gipec8Eeeu0xXdbba9frFj0=OqFfea0dXdd9vqai=hGuQ8kuc9pgc9s8qqaq=dirpe0xb9q8qiLsFr0=vr0=vr0dc8meaabaqaciaacaGaaeqabaqabeGadaaakeaaiiGacuWFYoGygaqcamaaBaaaleaacqaIYaGmaeqaaaaa@2F82@ as φ^=tan⁡−1(−β^2β^1)
 MathType@MTEF@5@5@+=feaafiart1ev1aaatCvAUfKttLearuWrP9MDH5MBPbIqV92AaeXatLxBI9gBaebbnrfifHhDYfgasaacH8akY=wiFfYdH8Gipec8Eeeu0xXdbba9frFj0=OqFfea0dXdd9vqai=hGuQ8kuc9pgc9s8qqaq=dirpe0xb9q8qiLsFr0=vr0=vr0dc8meaabaqaciaacaGaaeqabaqabeGadaaakeaaiiGacuWFgpGzgaqcaiabg2da9iGbcsha0jabcggaHjabc6gaUnaaCaaaleqabaGaeyOeI0IaeGymaedaaOWaaeWaaeaacqGHsisldaWcaaqaaiqb=j7aIzaajaWaaSbaaSqaaiabikdaYaqabaaakeaacuWFYoGygaqcamaaBaaaleaacqaIXaqmaeqaaaaaaOGaayjkaiaawMcaaaaa@3DE2@. The variance of φ^
 MathType@MTEF@5@5@+=feaafiart1ev1aaatCvAUfKttLearuWrP9MDH5MBPbIqV92AaeXatLxBI9gBaebbnrfifHhDYfgasaacH8akY=wiFfYdH8Gipec8Eeeu0xXdbba9frFj0=OqFfea0dXdd9vqai=hGuQ8kuc9pgc9s8qqaq=dirpe0xb9q8qiLsFr0=vr0=vr0dc8meaabaqaciaacaGaaeqabaqabeGadaaakeaaiiGacuWFgpGzgaqcaaaa@2E7C@ can be obtained using the delta method [16] and is shown to be:

var⁡(φ^)=β^22var⁡(β^1)+β^12var⁡(β^2)−2β^1β^2cov⁡(β^1,β^2)[β^12+β^22]2     (5)
 MathType@MTEF@5@5@+=feaafiart1ev1aaatCvAUfKttLearuWrP9MDH5MBPbIqV92AaeXatLxBI9gBaebbnrfifHhDYfgasaacH8akY=wiFfYdH8Gipec8Eeeu0xXdbba9frFj0=OqFfea0dXdd9vqai=hGuQ8kuc9pgc9s8qqaq=dirpe0xb9q8qiLsFr0=vr0=vr0dc8meaabaqaciaacaGaaeqabaqabeGadaaakeaacyGG2bGDcqGGHbqycqGGYbGCdaqadaqaaGGaciqb=z8aMzaajaaacaGLOaGaayzkaaGaeyypa0ZaaSaaaeaacuWFYoGygaqcamaaDaaaleaacqaIYaGmaeaacqaIYaGmaaGccyGG2bGDcqGGHbqycqGGYbGCdaqadaqaaiqb=j7aIzaajaWaaSbaaSqaaiabigdaXaqabaaakiaawIcacaGLPaaacqGHRaWkcuWFYoGygaqcamaaDaaaleaacqaIXaqmaeaacqaIYaGmaaGccyGG2bGDcqGGHbqycqGGYbGCdaqadaqaaiqb=j7aIzaajaWaaSbaaSqaaiabikdaYaqabaaakiaawIcacaGLPaaacqGHsislcqaIYaGmcuWFYoGygaqcamaaBaaaleaacqaIXaqmaeqaaOGaf8NSdiMbaKaadaWgaaWcbaGaeGOmaidabeaakiGbcogaJjabc+gaVjabcAha2naabmaabaGaf8NSdiMbaKaadaWgaaWcbaGaeGymaedabeaakiabcYcaSiqb=j7aIzaajaWaaSbaaSqaaiabikdaYaqabaaakiaawIcacaGLPaaaaeaadaWadaqaaiqb=j7aIzaajaWaa0baaSqaaiabigdaXaqaaiabikdaYaaakiabgUcaRiqb=j7aIzaajaWaa0baaSqaaiabikdaYaqaaiabikdaYaaaaOGaay5waiaaw2faamaaCaaaleqabaGaeGOmaidaaaaakiaaxMaacaWLjaWaaeWaaeaacqaI1aqnaiaawIcacaGLPaaaaaa@71D1@

An approximate (1 - α) 100% confidence interval is φ^±Zα2var⁡(φ^)
 MathType@MTEF@5@5@+=feaafiart1ev1aaatCvAUfKttLearuWrP9MDH5MBPbIqV92AaeXatLxBI9gBaebbnrfifHhDYfgasaacH8akY=wiFfYdH8Gipec8Eeeu0xXdbba9frFj0=OqFfea0dXdd9vqai=hGuQ8kuc9pgc9s8qqaq=dirpe0xb9q8qiLsFr0=vr0=vr0dc8meaabaqaciaacaGaaeqabaqabeGadaaakeaaiiGacuWFgpGzgaqcaiabgglaXkabbQfaAnaaBaaaleaadaWccaqaaiab=f7aHbqaaiabikdaYaaaaeqaaOWaaOaaaeaacyGG2bGDcqGGHbqycqGGYbGCcqGGOaakcuWFgpGzgaqcaiabcMcaPaWcbeaaaaa@3C36@, where Z_α/2 _is the (1 - α/2) 100% cut off point of the standard normal distribution.

The estimation of the amplitude can be obtained by A^=β^12+β^22
 MathType@MTEF@5@5@+=feaafiart1ev1aaatCvAUfKttLearuWrP9MDH5MBPbIqV92AaeXatLxBI9gBaebbnrfifHhDYfgasaacH8akY=wiFfYdH8Gipec8Eeeu0xXdbba9frFj0=OqFfea0dXdd9vqai=hGuQ8kuc9pgc9s8qqaq=dirpe0xb9q8qiLsFr0=vr0=vr0dc8meaabaqaciaacaGaaeqabaqabeGadaaakeaacuWGbbqqgaqcaiabg2da9maakaaabaacciGaf8NSdiMbaKaadaqhaaWcbaGaeGymaedabaGaeGOmaidaaOGaey4kaSIaf8NSdiMbaKaadaqhaaWcbaGaeGOmaidabaGaeGOmaidaaaqabaaaaa@374D@. The asymptotic variance of A^
 MathType@MTEF@5@5@+=feaafiart1ev1aaatCvAUfKttLearuWrP9MDH5MBPbIqV92AaeXatLxBI9gBaebbnrfifHhDYfgasaacH8akY=wiFfYdH8Gipec8Eeeu0xXdbba9frFj0=OqFfea0dXdd9vqai=hGuQ8kuc9pgc9s8qqaq=dirpe0xb9q8qiLsFr0=vr0=vr0dc8meaabaqaciaacaGaaeqabaqabeGadaaakeaaieaacuWFbbqqgaqcaaaa@2DCC@ can also be obtained using delta method and is shown to be:

var⁡(A^)=β^12var⁡(β^1)+β^22var⁡(β^2)+2β^1β^2cov⁡(β^1,β^2)β^12+β^22     (6)
 MathType@MTEF@5@5@+=feaafiart1ev1aaatCvAUfKttLearuWrP9MDH5MBPbIqV92AaeXatLxBI9gBaebbnrfifHhDYfgasaacH8akY=wiFfYdH8Gipec8Eeeu0xXdbba9frFj0=OqFfea0dXdd9vqai=hGuQ8kuc9pgc9s8qqaq=dirpe0xb9q8qiLsFr0=vr0=vr0dc8meaabaqaciaacaGaaeqabaqabeGadaaakeaacyGG2bGDcqGGHbqycqGGYbGCdaqadaqaaGqaaiqb=feabzaajaaacaGLOaGaayzkaaGaeyypa0ZaaSaaaeaaiiGacuGFYoGygaqcamaaDaaaleaacqaIXaqmaeaacqaIYaGmaaGccyGG2bGDcqGGHbqycqGGYbGCcqGGOaakcuGFYoGygaqcamaaBaaaleaacqaIXaqmaeqaaOGaeiykaKIaey4kaSIaf4NSdiMbaKaadaqhaaWcbaGaeGOmaidabaGaeGOmaidaaOGagiODayNaeiyyaeMaeiOCaiNaeiikaGIaf4NSdiMbaKaadaWgaaWcbaGaeGOmaidabeaakiabcMcaPiabgUcaRiabikdaYiqb+j7aIzaajaWaaSbaaSqaaiabigdaXaqabaGccuGFYoGygaqcamaaBaaaleaacqaIYaGmaeqaaOGagi4yamMaei4Ba8MaeiODayNaeiikaGIaf4NSdiMbaKaadaWgaaWcbaGaeGymaedabeaakiabcYcaSiqb+j7aIzaajaWaaSbaaSqaaiabikdaYaqabaGccqGGPaqkaeaacuGFYoGygaqcamaaDaaaleaacqaIXaqmaeaacqaIYaGmaaGccqGHRaWkcuGFYoGygaqcamaaDaaaleaacqaIYaGmaeaacqaIYaGmaaaaaOGaaCzcaiaaxMaadaqadaqaaiabiAda2aGaayjkaiaawMcaaaaa@6E7A@

An approximate (1 - α) 100% confidence interval is A^±Zα2var⁡(A^)
 MathType@MTEF@5@5@+=feaafiart1ev1aaatCvAUfKttLearuWrP9MDH5MBPbIqV92AaeXatLxBI9gBaebbnrfifHhDYfgasaacH8akY=wiFfYdH8Gipec8Eeeu0xXdbba9frFj0=OqFfea0dXdd9vqai=hGuQ8kuc9pgc9s8qqaq=dirpe0xb9q8qiLsFr0=vr0=vr0dc8meaabaqaciaacaGaaeqabaqabeGadaaakeaaieaacuWFbbqqgaqcaiabgglaXkabbQfaAnaaBaaaleaadaWccaqaaGGaciab+f7aHbqaaiabikdaYaaaaeqaaOWaaOaaaeaacyGG2bGDcqGGHbqycqGGYbGCcqGGOaakcuWFbbqqgaqcaiabcMcaPaWcbeaaaaa@3AE4@.

### Optimal Time and its Confidence Interval

Some may determine the optimal time by trying different partitions to the data where the variation looks cyclical. The simplest cyclical pattern is the sine wave with its associated parameters of amplitude, mean level (mesor), angle frequency, and phase angle (acrophase). This section will establish and construct an estimate and confidence interval of the day where the minimum hazard is achieved. The objective is to locate the optimal time for intervention, which is the time where the curve is at a minimum.

Since we know that cos π = -1, the optimum time must be when *π *= *ωt *+ φ^
 MathType@MTEF@5@5@+=feaafiart1ev1aaatCvAUfKttLearuWrP9MDH5MBPbIqV92AaeXatLxBI9gBaebbnrfifHhDYfgasaacH8akY=wiFfYdH8Gipec8Eeeu0xXdbba9frFj0=OqFfea0dXdd9vqai=hGuQ8kuc9pgc9s8qqaq=dirpe0xb9q8qiLsFr0=vr0=vr0dc8meaabaqaciaacaGaaeqabaqabeGadaaakeaaiiGacuWFgpGzgaqcaaaa@2E7C@, where *ω *= 2*π*/*τ*. Hence,

t^min⁡=π−φ^ω=τ[0.5−φ^2π]
 MathType@MTEF@5@5@+=feaafiart1ev1aaatCvAUfKttLearuWrP9MDH5MBPbIqV92AaeXatLxBI9gBaebbnrfifHhDYfgasaacH8akY=wiFfYdH8Gipec8Eeeu0xXdbba9frFj0=OqFfea0dXdd9vqai=hGuQ8kuc9pgc9s8qqaq=dirpe0xb9q8qiLsFr0=vr0=vr0dc8meaabaqaciaacaGaaeqabaqabeGadaaakeaacuWG0baDgaqcamaaBaaaleaacyGGTbqBcqGGPbqAcqGGUbGBaeqaaOGaeyypa0ZaaSaaaeaaiiGacqWFapaCcqGHsislcuWFgpGzgaqcaaqaaiab=L8a3baacqGH9aqpcqWFepaDdaWadaqaaiabicdaWiabc6caUiabiwda1iabgkHiTmaalaaabaGaf8NXdyMbaKaaaeaacqaIYaGmcqWFapaCaaaacaGLBbGaayzxaaaaaa@46C5@

The variance of t^min⁡
 MathType@MTEF@5@5@+=feaafiart1ev1aaatCvAUfKttLearuWrP9MDH5MBPbIqV92AaeXatLxBI9gBaebbnrfifHhDYfgasaacH8akY=wiFfYdH8Gipec8Eeeu0xXdbba9frFj0=OqFfea0dXdd9vqai=hGuQ8kuc9pgc9s8qqaq=dirpe0xb9q8qiLsFr0=vr0=vr0dc8meaabaqaciaacaGaaeqabaqabeGadaaakeaacuWG0baDgaqcamaaBaaaleaacyGGTbqBcqGGPbqAcqGGUbGBaeqaaaaa@327B@ is

var⁡(t^min⁡)=τ24π2var⁡(φ^)=τ2[β^22var⁡(β^1)+β^12var⁡(β^2)−2β^1β^2cov⁡(β^1,β^2)]4π2[β^12+β^22]2     (7)
 MathType@MTEF@5@5@+=feaafiart1ev1aaatCvAUfKttLearuWrP9MDH5MBPbIqV92AaeXatLxBI9gBaebbnrfifHhDYfgasaacH8akY=wiFfYdH8Gipec8Eeeu0xXdbba9frFj0=OqFfea0dXdd9vqai=hGuQ8kuc9pgc9s8qqaq=dirpe0xb9q8qiLsFr0=vr0=vr0dc8meaabaqaciaacaGaaeqabaqabeGadaaakeaafaqadeGabaaabaGagiODayNaeiyyaeMaeiOCai3aaeWaaeaacuWG0baDgaqcamaaBaaaleaacyGGTbqBcqGGPbqAcqGGUbGBaeqaaaGccaGLOaGaayzkaaGaeyypa0ZaaSaaaeaaiiGacqWFepaDdaahaaWcbeqaaiabikdaYaaaaOqaaiabisda0iab=b8aWnaaCaaaleqabaGaeGOmaidaaaaakiGbcAha2jabcggaHjabckhaYnaabmaabaGaf8NXdyMbaKaaaiaawIcacaGLPaaaaeaacqGH9aqpdaWcaaqaaiab=r8a0naaCaaaleqabaGaeGOmaidaaOWaamWaaeaacuWFYoGygaqcamaaDaaaleaacqaIYaGmaeaacqaIYaGmaaGccyGG2bGDcqGGHbqycqGGYbGCdaqadaqaaiqb=j7aIzaajaWaaSbaaSqaaiabigdaXaqabaaakiaawIcacaGLPaaacqGHRaWkcuWFYoGygaqcamaaDaaaleaacqaIXaqmaeaacqaIYaGmaaGccyGG2bGDcqGGHbqycqGGYbGCdaqadaqaaiqb=j7aIzaajaWaaSbaaSqaaiabikdaYaqabaaakiaawIcacaGLPaaacqGHsislcqaIYaGmcuWFYoGygaqcamaaBaaaleaacqaIXaqmaeqaaOGaf8NSdiMbaKaadaWgaaWcbaGaeGOmaidabeaakiGbcogaJjabc+gaVjabcAha2naabmaabaGaf8NSdiMbaKaadaWgaaWcbaGaeGymaedabeaakiabcYcaSiqb=j7aIzaajaWaaSbaaSqaaiabikdaYaqabaaakiaawIcacaGLPaaaaiaawUfacaGLDbaaaeaacqaI0aancqWFapaCdaahaaWcbeqaaiabikdaYaaakmaadmaabaGaf8NSdiMbaKaadaqhaaWcbaGaeGymaedabaGaeGOmaidaaOGaey4kaSIaf8NSdiMbaKaadaqhaaWcbaGaeGOmaidabaGaeGOmaidaaaGccaGLBbGaayzxaaWaaWbaaSqabeaacqaIYaGmaaaaaaaakiaaxMaacaWLjaWaaeWaaeaacqaI3aWnaiaawIcacaGLPaaaaaa@8DFA@

The asymptotic 95% confidence intervals will be based on the standard errors using an assumption of normality (t^min⁡±Zα2var⁡(t^min⁡))
 MathType@MTEF@5@5@+=feaafiart1ev1aaatCvAUfKttLearuWrP9MDH5MBPbIqV92AaeXatLxBI9gBaebbnrfifHhDYfgasaacH8akY=wiFfYdH8Gipec8Eeeu0xXdbba9frFj0=OqFfea0dXdd9vqai=hGuQ8kuc9pgc9s8qqaq=dirpe0xb9q8qiLsFr0=vr0=vr0dc8meaabaqaciaacaGaaeqabaqabeGadaaakeaadaqadaqaaiqbdsha0zaajaWaaSbaaSqaaiGbc2gaTjabcMgaPjabc6gaUbqabaGccqGHXcqScqqGAbGwdaWgaaWcbaWaaSGaaeaaiiGacqWFXoqyaeaacqaIYaGmaaaabeaakmaakaaabaGagiODayNaeiyyaeMaeiOCai3aaeWaaeaacuWG0baDgaqcamaaBaaaleaacyGGTbqBcqGGPbqAcqGGUbGBaeqaaaGccaGLOaGaayzkaaaaleqaaaGccaGLOaGaayzkaaaaaa@45CA@.

Bootstrapping also can be used to estimate the variability of the estimated function, and to provide information on whether certain features of the estimated function are true features of the data or just random noise [17,18].

### Motivating Application: Biological Timing of Breast Cancer Surgery

While seasonality affects us all, the menstrual cycle directly affects about 52% of the world's inhabitants. Each of the members of this small global majority spends about half of her life participating regularly and continuously in this powerful biological rhythm. Many diverse disease activities have been demonstrated to be affected by this cycle. Cancer is one of these [19-22]. It has been conjectured that menstrual stage at time of resection might affect breast cancer outcome. Recurrence of breast cancer disease may be affected by timing the surgery in relation to the menstrual cycle; therefore, the timing of surgery may be an important element that affects breast cancer outcome [23,24].

The timing of surgical intervention for breast cancer may have an influence on the outcome of these interventions [25,26]. Some studies have shown that patients who have surgery during the follicular phase (first 14 days of the cycle) have a higher recurrence rate than those treated during the luteal phase (second 14 days of the cycle). Other studies have shown that patients having surgery during the perimenstrual period (days 0–6 and 21–36) of the menstrual cycle had a quadrupled risk of recurrence and death compared with women operated upon during the middle (days 7 to 20) of their menstrual cycle [27,28]. Badwe et al. [29] stratified patients into groups containing patients whose LMP was 3–12 days before surgery and those who were operated on at other times. In contrast with other studies, they showed that overall and recurrence-free survivals were each enhanced for those who were resected during the luteal phase. Moreover, even the definitions of follicular and luteal phases were made based on different criteria in different institutions. Any time an article was published there, were subsequent letters to the editor or articles presenting contradictory results [30-32]. These discrepancies might be explained by the limited reliability of the menstrual history data, and the fact that these studies were retrospective. This has stimulated our interest in developing a more rigorous method to estimate the best time that can be recommended for surgical intervention based on a prospective study. The only way to really answer this question would be to perform a randomized controlled clinical trial.

The MA.5 study was a multi-center clinical trial conducted by the National Cancer Institute of Canada Clinical Trial Group (NCIC CTG) [33]. There were 262 pre-menopausal patients who had adequate data for LMP included in the study. All of them were eligible for the study, had normal menstruation, and regular period cycles (lasting between 21 and 35 days) [34]. The recurrence of breast cancer was confirmed with clinical or pathologic assessment, or both. Initial tumor size, status of the axillary lymph nodes, and other prognostic factors were assessed clinically and pathologically. Disease-free months were calculated from date of surgery to date of first relapse. The trial was activated December 1, 1989 and closed to accrual on July 31, 1993. The objective was to examine disease-free survival in relation to the timing of breast tumor excision during the menstrual cycle.

All analyses were conducted with SAS Version 9.1 and S-Plus for Windows Version 6.0. All tests were two-sided, and a level of α = 0.05 was used to determine a significant result. Product-limit survival curves were calculated by the method of Kaplan-Meier. The Cox proportional hazards model [15] was used to estimate the relative risk of relapse associated with timing of diagnostic surgery.

## Results

**Figure 1:**
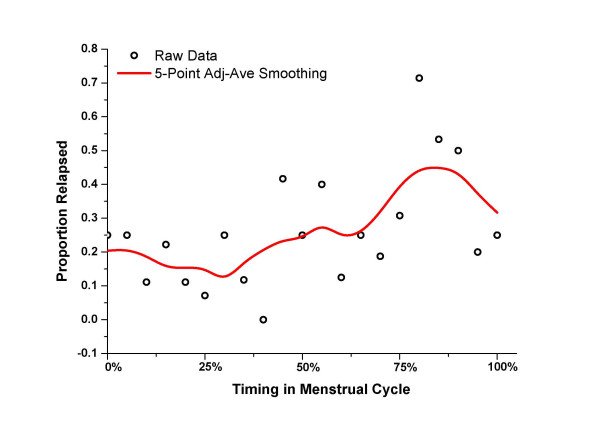
Proportion of recurrence according to day of the menstrual cycle at time of tumor excision.

The smoothed plot of the proportion of patients who relapsed (Figure 1) suggests that relapse is at a minimum when the tumor was excised during the first 1/4 of the menstrual cycle, gradually rising to a maximum during the last 1/4 cycle.

Multivariate analyses using the Cox Proportional Hazards model identified age, positive nodes, pathologic stage, and experimental treatment as the most significant factors related to disease free survival. The time of surgery within the menstrual cycle was a significant independent predictor of disease-free survival.

Assuming that the length of the cycle is 28 days for all women, and using back- transformation, we obtained the acrophase ϕ^=tan⁡−1(0.630.09)=1.42
 MathType@MTEF@5@5@+=feaafiart1ev1aaatCvAUfKttLearuWrP9MDH5MBPbIqV92AaeXatLxBI9gBaebbnrfifHhDYfgasaacH8akY=wiFfYdH8Gipec8Eeeu0xXdbba9frFj0=OqFfea0dXdd9vqai=hGuQ8kuc9pgc9s8qqaq=dirpe0xb9q8qiLsFr0=vr0=vr0dc8meaabaqaciaacaGaaeqabaqabeGadaaakeaaiiGacuWFvpGAgaqcaiabg2da9iGbcsha0jabcggaHjabc6gaUnaaCaaaleqabaGaeyOeI0IaeGymaedaaOWaaeWaaeaadaWccaqaaiabicdaWiabc6caUiabiAda2iabiodaZaqaaiabicdaWiabc6caUiabicdaWiabiMda5aaaaiaawIcacaGLPaaacqGH9aqpcqaIXaqmcqGGUaGlcqaI0aancqaIYaGmaaa@43A6@. Therefore, the minimum time of the curve is t^min⁡=14*(1−1.43.14)=7.7≈8 days
 MathType@MTEF@5@5@+=feaafiart1ev1aaatCvAUfKttLearuWrP9MDH5MBPbIqV92AaeXatLxBI9gBaebbnrfifHhDYfgasaacH8akY=wiFfYdH8Gipec8Eeeu0xXdbba9frFj0=OqFfea0dXdd9vqai=hGuQ8kuc9pgc9s8qqaq=dirpe0xb9q8qiLsFr0=vr0=vr0dc8meaabaqaciaacaGaaeqabaqabeGadaaakeaacuWG0baDgaqcamaaBaaaleaacyGGTbqBcqGGPbqAcqGGUbGBaeqaaOGaeyypa0JaeGymaeJaeGinaqJaeiOkaOYaaeWaaeaacqaIXaqmcqGHsisldaWcaaqaaiabigdaXiabc6caUiabisda0aqaaiabiodaZiabc6caUiabigdaXiabisda0aaaaiaawIcacaGLPaaacqGH9aqpcqaI3aWncqGGUaGlcqaI3aWncqGHijYUcqaI4aaocqqGGaaicqqGKbazcqqGHbqycqqG5bqEcqqGZbWCaaa@4D21@. The variance was estimated using equation (7) as 4.2 and hence the 95% confidence interval lies between 4 and 12 days.

**Figure 2:**
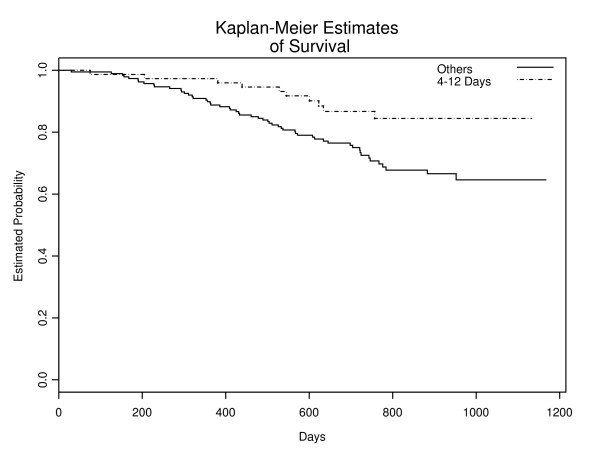
Relapse-Free Survival by Timing of Surgery: Proposed Definition.

Using this optimal interval, the disease recurred in 55 patients (30%) in the group were LMP was 0–3 and 13–40 days, whereas 10 patients (13%) developed disease in mid-cycle (4–12 days) group. Figure 2 shows a statistical significant difference in survival between these two groups (p = 0.0084). After controlling for age, positive nodes, pathologic stage, and arm, the menstrual phase at time of excision remain significantly associated with relapse-free survival (hazard ratio 0.425: CI. 0.214-0.845).

Since different comparisons had been made in the past based on retrospective analyses, it was of interest to compare results from approaches used in the past with the approach proposed in this study:

### Follicular vs. Luteal Stage Comparison

**Figure 3:**
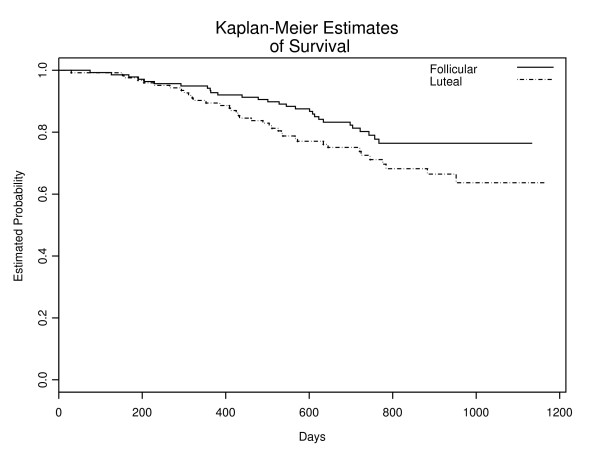
Relapse-Free Survival by Timing of Surgery: Senie's Definition.

The risk for recurrence differed between the two phases: 30% of patients developed recurrence after surgery in the luteal group compared with 20% in the follicular group. Figure 3 indicates a higher recurrence rate for patients with tumor excision during the luteal phase, but the differences in survival were not statistically significant (P = 0.07). However, after controlling for age, positive nodes, pathologic stage, and arm, the menstrual phase at time of excision was found to be significantly associated with relapse-free survival (p = 0.011; hazard ratio 1.93: CI. 1.16 – 3.19).

### Mid-cycle vs. Perimenstrual Stage Comparison

**Figure 4:**
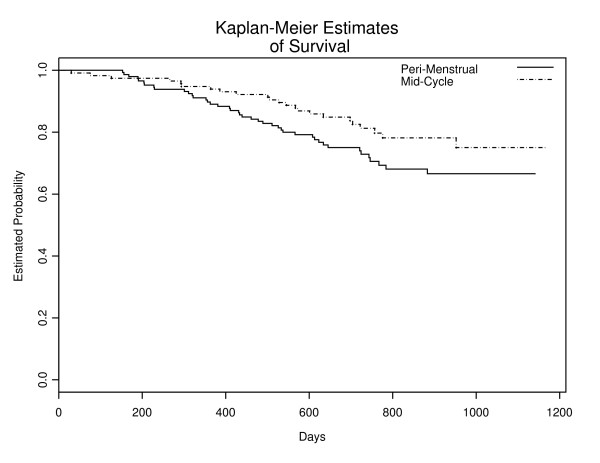
Relapse-Free Survival by Timing of Surgery: Hrushesky's Definition.

In this kind of menstrual interval, tumor recurred in 29% of Perimenstrual patients and in 20% of mid-cycle patients. Figure 4 indicates statistically insignificant difference in time to first recurred by phase (P = 0. 062). Based on Cox proportional hazards model, the time of surgery within the menstrual cycle is not an independent predictor of disease-free survival, p = 0.321 (hazard ratio 0.768: CI. 0.455 – 1.294).

## Discussion and Conclusion

In this paper we have described a method for modeling failure time data with an underlying biological rhythm. The advantage of adapting a cosinor model to proportional hazard model is its ability to model right censored data. We have presented a method to provide an estimate and confidence interval of the day where the minimum hazard is achieved.

The application of this technique to breast cancer data revealed that the optimal days for pre-resection incisional or excisional biopsy of 28-day cycle (i. e. the days associated with the lowest recurrence rate) are days 4–12. This represents the putative follicular phase for women with 28 to 36 day cycle duration and the luteal phase for those with the usual cycle length between 21 and 28 days. This is in agreement with the contention that disease recurrence and metastasis are more frequent and appear more rapidly in women who have had their initial breast cancer resection during days 0–6 and 21–36 of the menstrual cycle.

Most of studies designed to assess the efficacy of breast surgery in relation to the timing of the intervention during the menstrual cycle were retrospective. This article presents a prospective investigation of menstrual cycle operative timing. The proposed analytical technique is not limited to breast cancer data and may be applied to any biological rhythms linked to right censored data.

## Competing interests

We did not identify any situation that might be perceived as a conflict of interest.

## Authors' contributions

The authors contributed equally to this work.
